# KTED: a comprehensive web-based database for transposable elements in the Korean genome

**DOI:** 10.1093/bioadv/vbae179

**Published:** 2024-11-19

**Authors:** Jin-Ok Lee, Sejoon Lee, Dongyoon Lee, Taeyeon Hwang, Soobok Joe, Jin Ok Yang, Jibin Jeong, Jung Hun Ohn, Jee Hyun Kim

**Affiliations:** Department of Health Science and Technology, Graduate School of Convergence Science and Technology, Seoul National University, Seoul 13605, Republic of Korea; Precision Medicine Center, Seoul National University Bundang Hospital, Seongnam 13620, Republic of Korea; Department of Genomic Medicine, Seoul National University Bundang Hospital, Seongnam 13620, Republic of Korea; Korea Bioinformation Center, KRIBB, Daejeon 34141, Republic of Korea; Korea Bioinformation Center, KRIBB, Daejeon 34141, Republic of Korea; Korea Bioinformation Center, KRIBB, Daejeon 34141, Republic of Korea; Korea Bioinformation Center, KRIBB, Daejeon 34141, Republic of Korea; Department of Genomic Medicine, Seoul National University Bundang Hospital, Seongnam 13620, Republic of Korea; Precision Medicine Center, Seoul National University Bundang Hospital, Seongnam 13620, Republic of Korea; Department of Internal Medicine, Seoul National University Bundang Hospital, Seongnam 13620, Republic of Korea; Department of Internal Medicine, Seoul National University College of Medicine, Seoul 03080, Republic of Korea; Department of Genomic Medicine, Seoul National University Bundang Hospital, Seongnam 13620, Republic of Korea; Department of Internal Medicine, Seoul National University Bundang Hospital, Seongnam 13620, Republic of Korea; Department of Internal Medicine, Seoul National University College of Medicine, Seoul 03080, Republic of Korea

## Abstract

**Summary:**

Transposable elements (TEs), commonly referred to as “mobile elements,” constitute DNA segments capable of relocating within a genome. Initially disregarded as “junk DNA” devoid of specific functionality, it has become evident that TEs have diverse influences on an organism’s biology and health. The impact of these elements varies according to their location, classification, and their effects on specific genes or regulatory components. Despite their significant roles, a paucity of resources concerning TEs in population-scale genome sequencing remains. Herein, we analyze whole-genome sequencing data sourced from the Korean Genome and Epidemiology Study, encompassing 2500 Korean individuals. To facilitate convenient data access and observation, we developed a web-based database, KTED. Additionally, we scrutinized the differential distributions of TEs across five distinct common disease groups: dyslipidemia, hypertension, diabetes, thyroid disease, and cancer.

**Availability and implementation:**

https://snubh.shinyapps.io/KTED.

## 1 Introduction

Transposable elements (TEs) are DNA sequences that are able to move from one location to another within a genome (Wells and Feschotte 2020) and make up approximately half of the human genome (Tang *et al.* 2018). Most TEs in the genome are remnants of past insertion events and are now incapable of transposition. However, TEs such as Alu, Long Interspersed Nuclear Element 1 (LINE1), SINE-VNTR-Alu (SVA), and possibly Human Endogenous Retrovirus-K (HERV-K) elements, are still retrotranspositionally competent in the human genome and capable of creating new insertions (Rishishwar *et al.* 2015). TE insertions can influence the expression or structure of a gene by supplying alternative splicing and transcription binding sites, contributing to diverse diseases such as neurodegenerative, autoimmune, genetic disorders, and cancers (Payer and Burns 2019, Clayton *et al.* 2020, Jönsson *et al.* 2020, Chenais 2022, Fueyo *et al.* 2022). For example, TEs can influence oncogenesis through a prevalent phenomenon termed onco-exaptation where cryptic regulatory elements within TEs can be epigenetically reactivated in cancer (Jang *et al.* 2019). The dysregulation of TE expression or TE activation, particularly of HERV-K, leads to neuronal death and neurodegeneration by tauopathies in human brains (Evering *et al.* 2023). Hundreds of different Alu insertions have been identified in areas linked to common diseases such as endocrine, cardiovascular, psychiatric, and autoimmune diseases, suggesting that Alu may be a variant that influences disease risk (Payer *et al.* 2017). Advancements in whole-genome sequencing (WGS) are enabling more precise analysis of TEs’ contributions to the human genome at a population level (Gardner *et al.* 2017, Chu *et al.* 2021). Several studies have been conducted have provided comprehensive resources on polymorphic TEs within the human genome, aiding in exploring the diversity of TEs and their association with various phenotypes. The 1000 Genomes Project (1KGP) carried out an extensive analysis of TE variations, including over 20 000 polymorphic TEs across 2504 genomes (Sudmant *et al.* 2015, Gardner *et al.* 2017). Watkins *et al.* conducted research on the 296 genomes from 142 different populations (Watkins *et al.* 2020). Recent studies have been conducted on the genomes of 2998 Chinese individuals (NyuWa) (Niu *et al.* 2022). Kojima *et al.* analyzed TEs in 1KGP and 1235 individuals from BioBank Japan (BBJ) using their self-developed tool, revealing TEs as drivers of population differences and pathogenesis (Kojima *et al.* 2023). The analysis of TEs from 1000 Swedish individuals in the SweGen dataset also highlighted the potential clinical significance in the diagnosis of rare diseases (Bilgrav Saether *et al.* 2023). However, comprehensive resources for polymorphic TEs in the human genome are still limited and research on East Asians remains relatively scarce compared to that on Europeans. Notably, there are no studies specifically targeting Koreans.

In this study, we aim to explore the distribution of TEs in a cohort study of 2500 Koreans, encompassing both healthy individuals and those with common complex diseases such as dyslipidemia, hypertension, diabetes, thyroid disease, and cancer. By comparing the identified TEs in the Korean population with those from the 1000 Genomes Project and the NyuWa dataset, we can contrast and analyze the unique TE distribution characteristics of Koreans against other racial and ethnic groups. The primary goal is to develop a user-friendly web-based database for this TEs distribution, facilitating easy access and analysis for researchers. This study is expected to provide novel insights into the role of TEs by offering information on the distribution of TEs in Koreans.

## 2 Methods

### 2.1 Subjects

**Figure 1.**
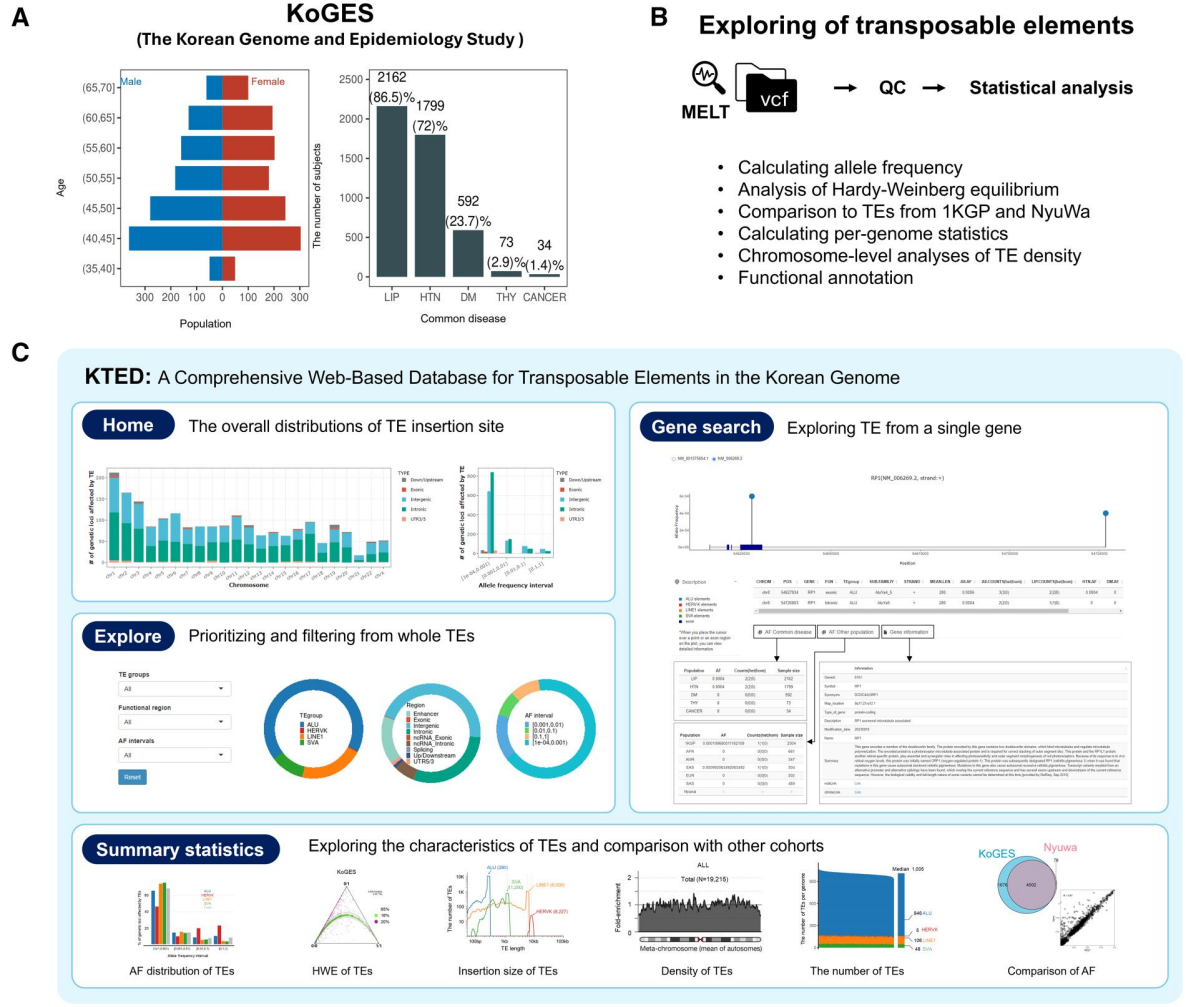
Overview of our study. (A) The genomic data and clinical information of the KoGES cohort. (B) The workflow of Transposable elements (TEs) detection using MELT and the analysis of characteristic of TEs. (C) The KTED web-server.

TEs were analyzed based on WGS data obtained from 2500 individuals collected from The Korean Genome and Epidemiology Study (KoGES). KoGES is a large cohort study that recruited randomly selected men and women aged 40–69 (52 ± 8.27) years from 2001 to 2003 designed to identify the genetic and environmental etiologies of common complex diseases in the Korean population. Participants were examined using epidemiological surveys, physical examinations, and laboratory tests at baseline. A medical examination and a questionnaire-based interview were administered every 2 years to compile longitudinal data (Kim *et al.* 2017). In this study, information about disease diagnoses at baseline was available for five common disorders: dyslipidemia, hypertension, diabetes, thyroid disease, and cancer of any type. For hypertension, diabetes, and thyroid disease, the classification also included the occurrence or persistence of these diseases during the follow-up period. In the cohort, dyslipidemia was the most common condition, diagnosed in 2162 individuals, followed by hypertension (1799), diabetes (592), and thyroid disorders (73). Cancer of any type was the least common, with 34 individuals (Fig. 1A).

### 2.2 Whole genome sequencing

The WGS data from the KoGES cohort were generated using the Illumina NovaSeq 6000 platform (coverage mean ∼40.0×). After preprocessing, including PCR deduplication and removal of adapter sequences and low-quality bases (below Q30) with FastQC v0.11.9, Cutadapt v3.6, and GATK v4.2.4.1, the sequence reads were aligned to the reference genome (GRCh38) utilizing BWA-MEM v0.7.15 (Vasimuddin *et al.* 2019) (Fig. 1A).

### 2.3 Generation of TEs and functional annotations

Individual VCFs were generated by MELT v2.2.2 (Gardner *et al.* 2017) from BAM files, which detect a genome-wide range of Alu, LINE1, SVA, and HERV-K insertions. Variants were selected following criteria to obtain a high-quality call TE call: (i) MELT ASSESS score ≥3; (ii) split reads >2; and (iii) VCF FILTER columns be PASS. The allele frequency of TEs was calculated as the ratio of allele count to allele number. The allele count was the number of alternate alleles in the genotype field across all the samples, and the allele number was the total number of alleles in called genotypes. The allele frequencies (AF) were divided into five bins: 0 ≤ AF < 0.0001, 0.0001 ≤ AF < 0.001, 0.001 ≤ AF < 0.01, 0.01 ≤ AF < 0.1, and 0.1 ≤ AF < 1 and the proportions of TEs in each AF frequency interval was calculated. To assess the functional implications of TE insertions within genomic regions, we conducted gene-based annotation analysis using ANNOVAR (Wang *et al.* 2010) (Fig. 1B). TEs were annotated as “Enhancer” in intron or intergenic regions from GeneHancer database v5.20 (Fishilevich *et al.* 2017) employing BEDtools v2.28.0 (Quinlan and Hall 2010), following the methods described in a previous study (Niu *et al.* 2022).

### 2.4 Comparison KoGES TEs with 1KGP and NyuWa

To compare the TEs generated by the high-coverage 1KGP (Sudmant *et al.* 2015) and NyuWa (Niu *et al.* 2022), we downloaded the GRCh38 version call set from IGSR (Fairley *et al.* 2020) and HMEID (http://bigdata.ibp.ac.cn/HMEID/), respectively. The comparison of AF between cohorts was based on the approach proposed in the previous study (Collins *et al.* 2020, Niu *et al.* 2022). The approach for comparing AF across cohorts was as follows: We first selected candidate variants within 300 bp of the TE discovery regions (breakpoints) for each cohort using “window” function from BEDtools v2.28.0 (Quinlan and Hall 2010). The 300 bp cutoff criterion was established based on the gnomAD-SV analysis study. Selected TEs were then visualized using Venn diagrams. AF correlation analysis was performed only for identical TE types at the same locations between two cohorts using R package.

### 2.5 Analysis of disease-associated TEs

We conducted a comprehensive analysis for five diseases: dyslipidemia, hypertension, diabetes, thyroid disease, and cancer. For each disease, we divided the subjects into two groups: those with the disease (case group) and those without the disease (control group). We then performed either a chi-square test or Fisher’s exact test to compare the AF between these groups for each genetic variant. To account for multiple testing, we applied the False Discovery Rate (FDR) method to adjust the *P*-values. We considered variants with an adjusted *P*-value <0.05 as statistically significant.

### 2.6 Analysis of TEs’ characteristics

To check the distribution and length of TEs throughout the genome, we used the method described by Collins *et al.* (https://github.com/talkowski-lab/gnomad-sv-pipeline/tree/master/gnomad_sv_manuscript_code) (Collins *et al.* 2020). For chromosome-level analysis for TE density, we segmented into consecutive 100 kb bins and count the TEs per bins except the centromere. After smoothing the TE counts for each TE type and normalizing the results to identify enrichment patterns in different chromosomes. To evaluate the genotype distributions of each TE under the null expectations set by the Hardy-Weinberg equilibrium (HWE), we performed an exact test using the “HWExactStats” function in the R package HardyWeinberg 1.7.8 (Graffelman 2015).

### 2.7 Statistical analysis

All statistical analyses in this study were performed using R (version 4.2.3, http://CRAN. R-project.org/).

## 3 Results

### 3.1 TE populations

After site quality filtering, a total of 19 212 TEs were kept, including 13 137 Alu, 3981 LINE1, 2064 SVA, and 30 HERV-K. On average, 1005 TEs were detected per genome (Supplementary Table S1). The number of TEs detected in Koreans was similar to that observed in other cohort studies. The NyuWa dataset had an average of 1236 TEs per genome and 1040 TEs in the 1KGP dataset (Niu *et al.* 2022). About 70% of the total TEs are very rare (AF < 0.001) with approximately 52% located in intergenic regions and around 45% in intronic regions (Supplementary Fig. S1). In the exon regions, 93 TEs were identified, including 55 Alu, 2 HERV-K, 14 LINE1, and 22 SVA. Among these, 71 (76%) were confirmed to be rare variants with a frequency range (AF < 0.001). Among TEs, Alu were the most abundant, with 58 Alu Y subfamilies identified. Examination of TE subfamilies revealed distributions of active Alu and LINE1 TEs consistent with previous observations in humans. For instance, AluYa5 and AluYb8 were identified as the two most abundant Alu subfamilies, indicating their high retrotransposition activity in modern humans (Supplementary Fig. S2). In analysis of AF across various diseases, we observed a statistically significant association only in thyroid disease. Specifically, we identified a significant LINE1 insertion (chr5:159924192, adjusted *P*-value: 0.0022) in the intronic region of the *ADRA1B* gene.

### 3.2 Characteristics of TE

When compared to 1KGP and NyuWa, KoGES was found to have discovered new TEs loci do not present in the other cohorts, comprising 8241 ALU, 14 HERV-K, 2862 LINE1, and 1583 SVA loci (Supplementary Fig. S3B). When selecting candidate variants for comparison with other cohorts, we observed that KoGES showed insertions at a more diverse range of similar positions. Meanwhile, the correlation of AF between the cohorts was confirmed to be significantly high at 0.89 and 0.97, respectively (Supplementary Fig. S3C). Using HWE as a rough proxy of genotyping accuracy, we found that about 80% of autosomal TE sites had high genotyping accuracy (Supplementary Fig. S3D). At the chromosome-level density, the distribution of TEs showed a similar distribution across the genome, except for SVA and LINE1 elements, Notably, LINE1 exhibited a relatively higher frequency in centromeric regions compared to telomeric regions (Supplementary Fig. S4).

### 3.3 Database infrastructure

The KTED was constructed based on the R-based shiny web application framework packages, and it contains JavaScript, HTML and CSS. KTED is a comprehensive TE database featuring an intuitive interface and providing access to four main sections: “Home,” “Gene search,” “Explore” and “Summary” (Fig. 1). At “Home,” users can explore the overall distribution and AFs of four types of TEs across the entire genome (Supplementary Fig. S5). The “Gene search” menu allows users to explore the positions of TEs within genes of interest and observe the AF of these TEs in five different disease groups or the entire population. In “Explore,” users can investigate the distribution of TEs based on their selected preferences. Additionally, in “Summary,” users can view the summary of the exploration of TEs.

#### 3.3.1 Gene search

The “Gene Search” menu is designed to identify the insertion locations of TEs within selected gene regions. Information on gene structures and transcript IDs was obtained from “GCF_000001405.40_GRCh38.p14_genomic.gtf”, with only transcript IDs starting with NM_* selected for visualization. Gene information was sourced from NCBI. When users select a gene of interest from the search menu, they can view one gene structure diagram based on the unique identifiers of the transcripts expressed from that gene and one table. For each transcript ID, users can visualize the TE insertion locations and AF in a lollipop plot. Hovering over the dot points provides brief information, while more detailed information, including genomic location, gene-based annotation, TE group, TE information (length, subfamily), and AF, can be found in the table. By selecting a row in the table (i.e. one variant) and clicking the “AF: Common disease,” users can view the AF and subject count for five disease groups for the selected variant. In the “AF: Other population” tab, users can view the AF 1KGP and NyuWa populations. When clicking “Gene information,” basic gene functions are provided for user convenience, accompanied by gene information and immediate access to NCBI (Supplementary Fig. S6).

#### 3.3.2 Explore

The “Explore” delivers an exhaustive table depicting the TEs deployed for the study. To enhance user convenience, two functions are provided. Firstly, users can filter by TE group, genomic regions with TE insertions, and AF interval criteria. Secondly, the table is equipped with a header-based sorting mechanism and a search function to expedite the process of searching and identifying desired information (Supplementary Fig. S7).

#### 3.3.3 Summary

In the Summary menu, users can view a summary of the population composition of KoGES and the distribution, length, and subfamily distribution of Alu and LINE1 found in this population. Additionally, genome-based density of TEs, and HWE evaluation results are available. The results of comparative analyses of AF between different cohorts (1KGP and NyuWa) also found in this menu.

### 3.4 Case studies

The *RP1* gene is located on chromosome 8 and encodes a protein associated with photoreceptor microtubule-related proteins. This protein plays an essential role in the proper stacking of outer segment discs in rod photoreceptors and is closely related to retinitis pigmentosa. The *RP1* c.5797C > T:p.(Arg1933*) variant is relatively common in Koreans and East Asians, and while it does not cause macular dystrophy on its own, it can lead to macular dystrophy when occurring in conjunction with Alu insertion in *RP1* exon 4 (Nikopoulos *et al.* 2019). Alu insertion in *RP1* was not found in 1KGP, except among East Asians, nor in the gnomAD SV 2.1 and NyuWa cohorts. It was identified in only one out of 504 individuals in the East Asian cohort of 1KGP and in three out of 2500 individuals in a Korean cohort study. This information from the KTED provides valuable insights into ophthalmic disease research. We have identified a rare LINE1 insertion in exon 7 of the *LMBRD2* gene (chr5:36124196), which was observed in only two out of 2500 Korean individuals. *LMBRD2* involved in adrenergic receptor signaling pathway and associated with autosomal dominant disorder characterized by onset of motor and speech delay in early childhood (Malhotra *et al.* 2021). LINE1 insertions in exonic regions are known to potentially affect gene expression (Kazazian *et al.* 1988). Moreover, the LMBRD2 protein region where this LINE1 insertion occurred (Q68DH5: 250–274) has a high AlphaMissense pathogenicity score (>0.9), suggesting that this insertion may cause structural issues in the protein. Considering these factors, this LINE1 insertion in exon could have a significant impact on the function of the LMBRD2 protein, potentially affecting individuals carrying this variant.

Additionally, we identified TE insertions as heterozygous alleles in the exonic regions of several genes associated with Mendelian disorders. Notably, an SVA insertion was found in the *KIAA0586* (chr14:58547976) gene in two out of our cohort. Biallelic mutations in this gene are known to lead to Joubert syndrome and retinal dystrophy (Bachmann-Gagescu *et al.* 2015). Furthermore, we identified Alu insertions in the *MYH6* (chr14:23393776) and *HMGCL* (chr1:23820555) genes, each observed in only one individual. The *MYH6* gene encodes the alpha heavy chain subunit of cardiac myosin, a critical component of the cardiac contractile apparatus. Mutations in this gene have been associated with a spectrum of cardiac disorders, including hypertrophic and dilated cardiomyopathy, as well as atrial septal defects (Carniel *et al.* 2005). *HMGCL* encodes a protein that plays a crucial role in the ketogenesis pathway and leucine metabolism. The mutations of *HMGCL* gene cause HMG-CoA lyase deficiency, which is an autosomal recessive inborn error of metabolism (Pié *et al.* 2007). These findings suggest potential functional implications of TE insertions within these disease-associated genes, warranting further investigation into their impact on gene function and disease pathogenesis. Future studies should focus on elucidating the specific functional consequences of these TE insertions in the context of their respective associated disorders.

We identified a previously known pathogenic TE insertion in the 3′UTR of the *FKTN* gene (chr9:10563963) as a heterozygous allele in seven individuals. As previously reported, a SVA insertion in *FKTN’*s 3′-UTR is associated with Fukuyama muscular dystrophy. This insertion creates a cryptic splice site 116 bp upstream of the stop codon in exon 10, resulting in an additional exon 11 (Taniguchi-Ikeda *et al.* 2011). Pathogenic TE insertions are associated with various hereditary cancers, including hereditary breast and ovarian cancer syndrome (*BRCA1*, *BRCA2*, and *BARD1*) (Qian *et al.* 2017); Lynch syndrome (*MLH1*) (Solassol *et al.* 2019); and colorectal cancer (*APC*) (Cajuso *et al.* 2019). While these disease-causing TE insertions have been associated with exonic regions, intronic insertions are also found to have significant impacts. A previous study identified an SVA insertion in *BRCA1* intron 2 in a tumor, which led to reduced *BRCA1* expression (Walsh *et al.* 2021). Alu insertion in *MLH1* intron 7 results in aberrant splicing and transcription, causing Lynch syndrome (Li *et al.* 2020). In the KoGES data, we have identified rare intronic TE insertions in those genes associated with these hereditary cancer syndromes. These findings highlight the potential importance of noncoding structural variants in disease etiology and underscore the need for comprehensive genomic analyses that can detect such variants.

## 4 Discussion

Our study utilized clinical and genomic data obtained from the KoGES cohort, a national research project conducted by the National Institute of Health under the Korea Disease Control and Prevention Agency. Due to the nature of this data, direct experimental validation of individual genetic variants is practically difficult. MELT is a tool for detecting polymorphic inherited insertions and has been widely adopted by several projects, such as 1KGP and the gnomAD-SV database. In 1KGP, TE insertion sites discovered by MELT were extensively validated using PCR-based methods. Furthermore, Niu *et al.* validated MELT results in the NyuWa dataset and the results showed considerably low error rates: Alu 4.55%, LINE1 4.08%, SVA 6.45%, and HERV-K 23.08% (Niu *et al.* 2022). Considering these previous findings, we followed the method proposed by the previous study and applied stringent filtering criteria to the initial MELT detection results: (ⅰ) MELT ASSESS score ≥3; (ⅱ) split reads >2; and (ⅲ) VCF FILTER columns be PASS (Niu *et al.* 2022). Although we were unable to perform direct experimental validation, we believe this approach has contributed to enhancing the reliability of our research findings.

TE insertions and deletions lead to polymorphisms, showing substantial geographic differentiation, with numerous group-specific polymorphic insertions reported (Rishishwar *et al.* 2015). Given these observations, comprehensive analyses of TEs across diverse populations and disease states are necessary. However, relatively more research has been conducted on insertions in disease causation and human population history compared to deletions. In this study, we also focused on TE insertions distribution using the MELT tool. Recent studies have begun to address TE deletions (Autio *et al.* 2021, Cuenca-Guardiola *et al.* 2023), and we plan to investigate this aspect in our future research.

Wildschutte *et al.* identified a total of 46 HERV-K insertions using two methods: first, using RetroSeq to detect read pair signatures; second, mining unmapped reads for LTR-genome junctions. They then estimated AF for 27 of these insertions, along with 13 additional known polymorphic sites, across both 1KGP and Human Genome Diversity Project (HGDP) cohorts (Wildschutte *et al.* 2016). To compare the loci of Korean HERV-K with those reported in this study, we selected variants within 300 bp of the HERV-K discovery regions for each study. From the KoGES dataset, 17 regions out of 30 were selected, and from the previous study, 9 regions out of 46 were selected. While the positions of the variants did not match exactly, we found that the base differences between each corresponding variant ranged from 1 to 12 base pairs, indicating close proximity (Supplementary Table S2).

HWE analysis of TEs in KoGES revealed that the majority of loci conform to HWE expectations, suggesting good overall genotyping quality. However, we identified a subset of loci that deviate from equilibrium. These deviations, while potentially indicating genotyping errors, may also represent significant biological phenomena. Future research should focus on conducting more detailed analyses of these outlier loci to elucidate potential selection pressures or evolutionary mechanisms at play. This approach may uncover previously unrecognized patterns of TE activity, contributing to a more comprehensive understanding of their role in genomic evolution.

We have identified *ADRA1B* as a significant gene associated with thyroid disease. This gene encodes the alpha-1B adrenergic receptor, which has been previously reported to be linked to papillary thyroid carcinoma (Zhong *et al.* 2022). While these results suggest a possible connection between retrotransposon activity and thyroid pathophysiology, it is important to note that further research is essential to validate and elucidate the significance of this discovery in thyroid disease mechanisms.

In this study, we identified TEs inserted into genes associated with diseases, including those related to known pathogenic TEs and Mendelian inheritance. A limitation of our study was the lack of detailed clinical information on the subjects, which prevented us from accurately determining the direct association between these insertions and diseases. However, we expect that the database constructed through this research will serve as an important reference for future studies on related diseases.

## 5 Conclusion

This study analyzed the distribution and frequency of four types of TEs in a cohort of 2500 Koreans and identified the distribution of TEs in common complex diseases. These databases offer new perspectives on the relationship between TEs and diseases and contribute to a better understanding of disease research, diagnosis, prevention, and treatment in Koreans. Additionally, the KTED web-based database enhances researchers’ ability to easily access and analyze TE prevalence and distribution. Future advances in TE research, combined with the outcomes presented in this study, promise to contribute to the study and treatment of diseases in the Korean population.

## Supplementary Material

vbae179_Supplementary_Data

## Data Availability

The data underlying this article are available at https://snubh.shinyapps.io/KTED.
