# Supplementary material for: KTED: a comprehensive web-based database for transposable elements in the Korean genome
Source: Bioinform Adv. 2024 Nov 19;4(1):vbae179. doi: 10.1093/bioadv/vbae179 (PMC11652267; doi:10.1093/bioadv/vbae179)
Supplement: vbae179_Supplementary_Data [file vbae179_supplementary_data.docx]

**Suppl Table 1. TE discovery**

| TE types | Total sites | Per subjects | |
| --- | --- | --- | --- |
|  |  | Mean | SD |
| ALU | 13,137 | 845.8 | 20.6 |
| LINE1 | 3,981 | 106.2 | 7 |
| SVA | 2,064 | 48.22 | 5.2 |
| HERV-K | 30 | 4.81 | 1.2 |
| Total | 19,212 | 1,005 | 23.9 |

**Suppl Table 2. Comparison of HERV-K Insertions in KoGES and Wildschutte et al Studies**

| KoGES (hg38) | Wildschutte et al. (hg38) | Wildschutte et al. (hg19) |
| --- | --- | --- |
| chr1:111259975 | chr1:111259970 | chr1:111802592 |
| chr5:81146453 | chr5:81146447 | chr5:80442266 |
| chr6:16004632 | chr6:16004628 | chr6:16004859 |
| chr6:160849860 | chr6:160849867 | chr6:161270899 |
| chr6:160849863 | chr6:160849867 | chr6:161270899 |
| chr6:160849866 | chr6:160849867 | chr6:161270899 |
| chr6:160849869 | chr6:160849867 | chr6:161270899 |
| chr6:160849872 | chr6:160849867 | chr6:161270899 |
| chr10:99256377 | chr10:99256365 | chr10:101016122 |
| chr11:60682422 | chr11:60682417 | chr11:60449890 |
| chr12:123581933 | chr12:123581930 | chr12:124066477 |
| chr12:123581935 | chr12:123581930 | chr12:124066477 |
| chr12:43919853 | chr12:43919854 | chr12:44313657 |
| chr12:43919855 | chr12:43919854 | chr12:44313657 |
| chr12:43919857 | chr12:43919854 | chr12:44313657 |
| chr12:43919859 | chr12:43919854 | chr12:44313657 |
| chr19:29364877 | chr19:29364874 | chr19:29855781 |


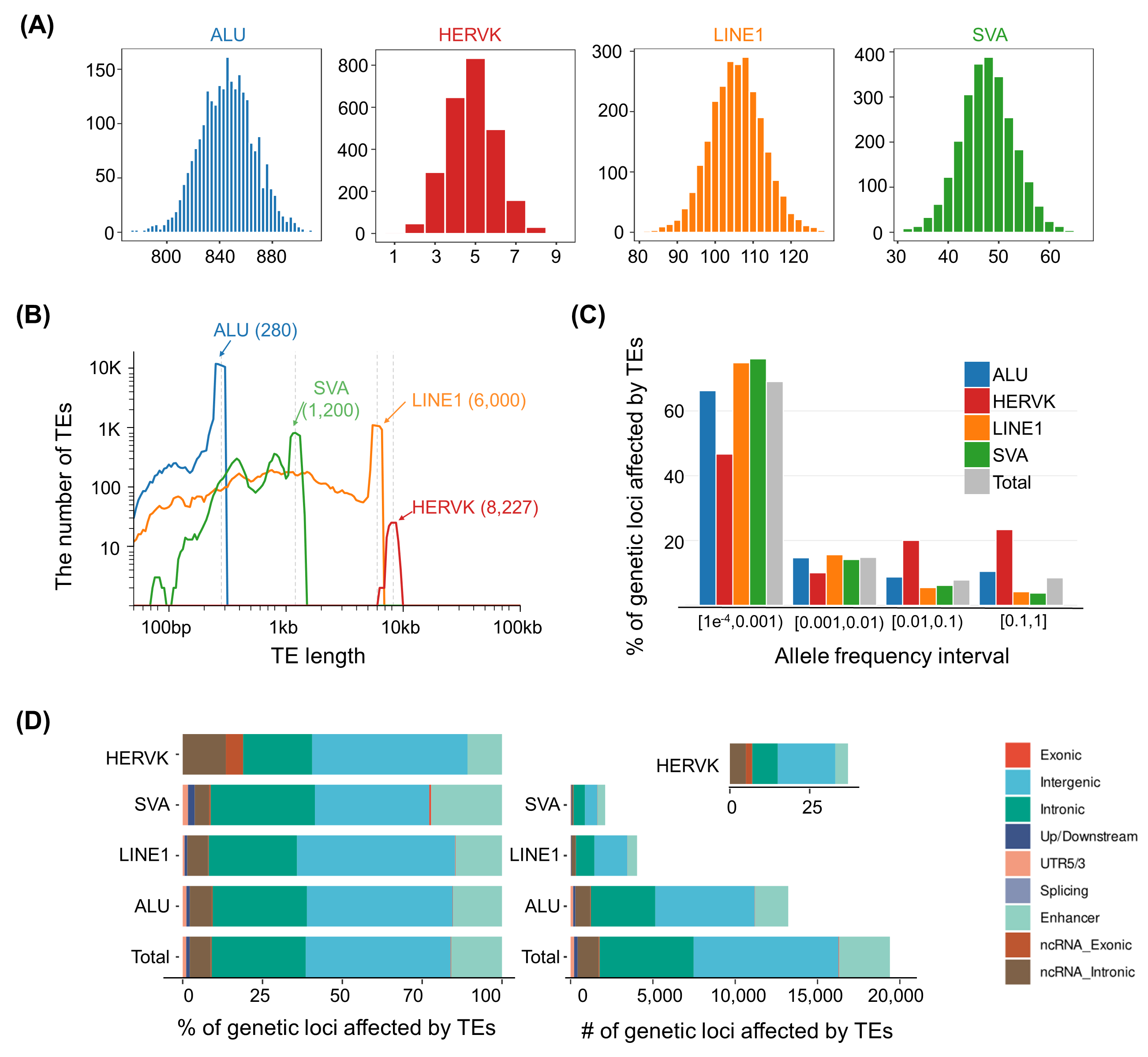


**Suppl Figure 1. TEs by MELT in KoGES.** (A) Histograms of the number of TEs per individual genomes (B) Insert size of TEs. The x-axis coordianates of peaks were annotated (C) Distribution of allele frequency of TEs of four type: Alu, Line1,SVA and HERV-K. The allele frequencies were cut in to five bins: 0 ≤ AF < 0.0001, 0.0001 ≤ AF < 0.001, 0.001 ≤ AF < 0.01 0.01 ≤ AF < 0.1 and 0.1 ≤ AF < 1 and the proportions of TEs in each AF frequency interval was calculated. (D) Distribution of genes affected by TEs of four types. “Total” combined the four types of TEs.:(left) cumulative proportion, and (right) cumulative number.

**
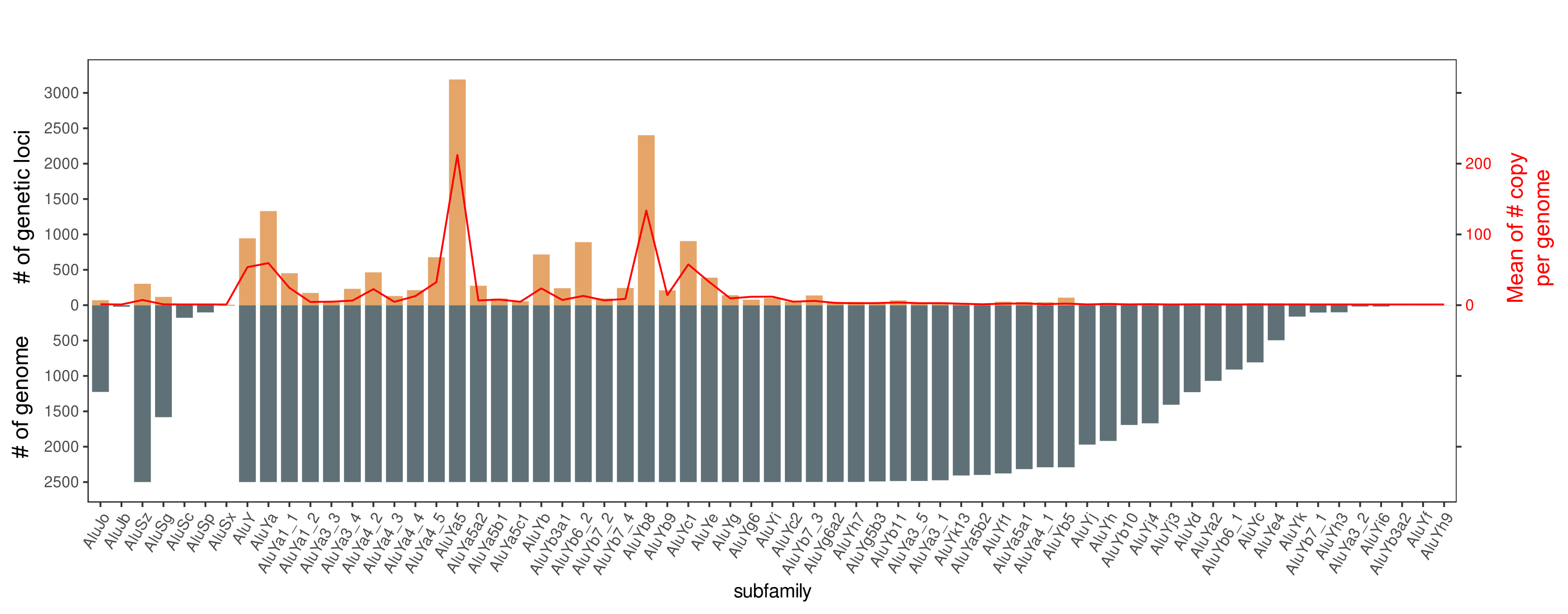
**

**Suppl Figure2. The distribution of Alu subfamiliy**. Bar plot shows the frequency of various Alu subfamilies in this study. The upper bar represnet the number of locus where the subfamily is located, while the lower bar indicated the number of detection in KoGES population. Thre red line denotes the number of the copies each individual has.


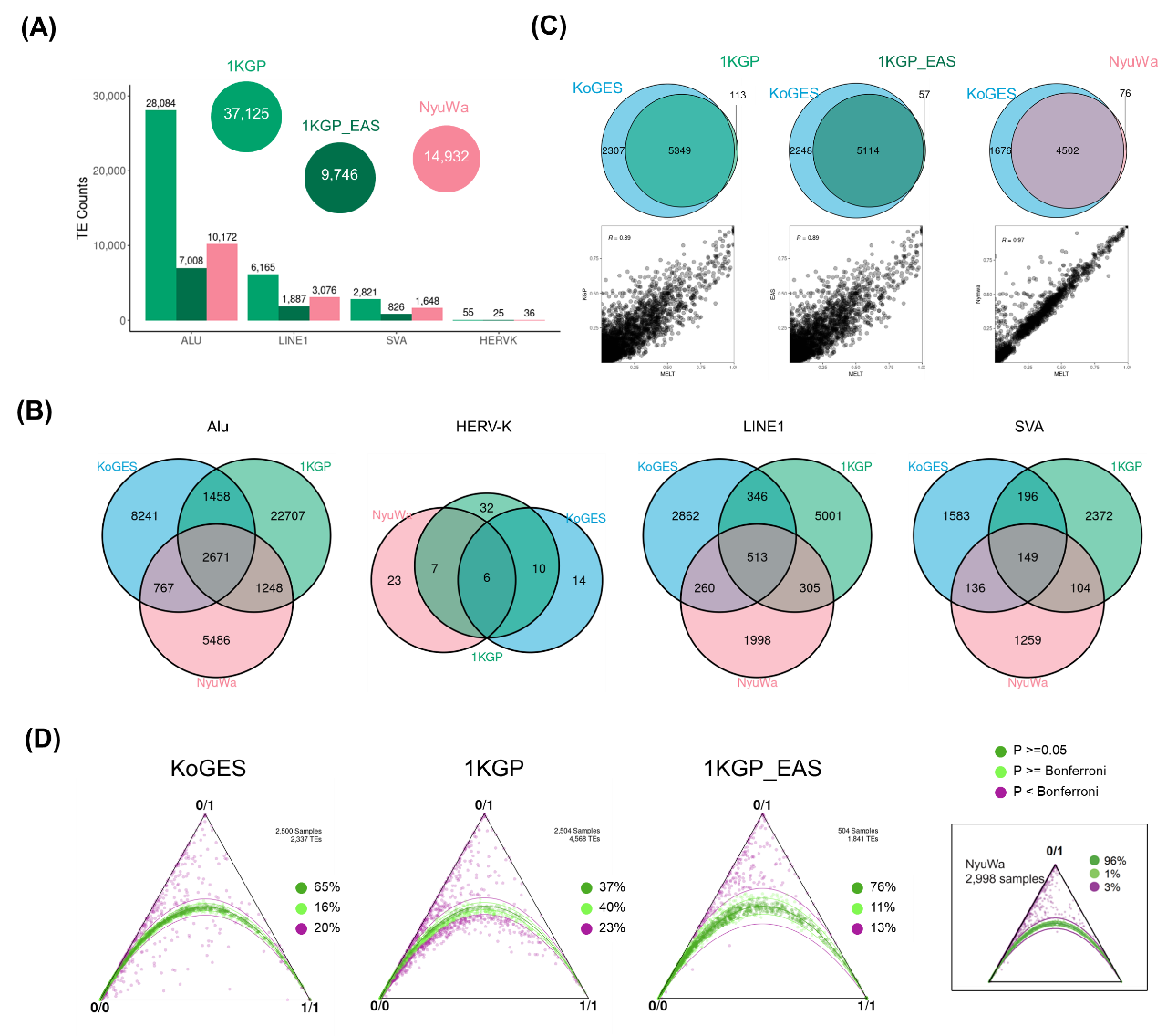


**Suppl Figure 3**. Comparing KoGES with other TE databases: 1KGP and NyuWa cohort. (A) The bar plot shows the detected TEs on each cohort (1KGP, East Asian in 1KGP and NyuWa). (B) Venn diagrams show the comparison of all TE call set for each sugroups across cohorts. (C) The venn diagrams show the comparison of TE call set in KoGES and other cohorts for selected insertions for AF correlation allele frequency (AF) correlation analysis. Correlation of AF for TEs found in both each cohort and this study. (D) Hardy-Weinberg Equilibrium (HWE) statistics was computed for TEs contained at least one heterohomozygote, detected in this study on KoGES, 1KGP and East Asian in 1KGP. The HWE results in the box were obtained from a previous paper on the NyuWa cohort (Xy and He. et al). Vertex labels are three types of genotypes: 0/0 represents homozygous reference; 0/1 represents heterozygous; and 1/1 represents homozygous alternatives. Points represent MEIs in the indicated sample set, shaded with P values by exact test. The distance of a point to a vertex is the fraction of samples with that genotype.


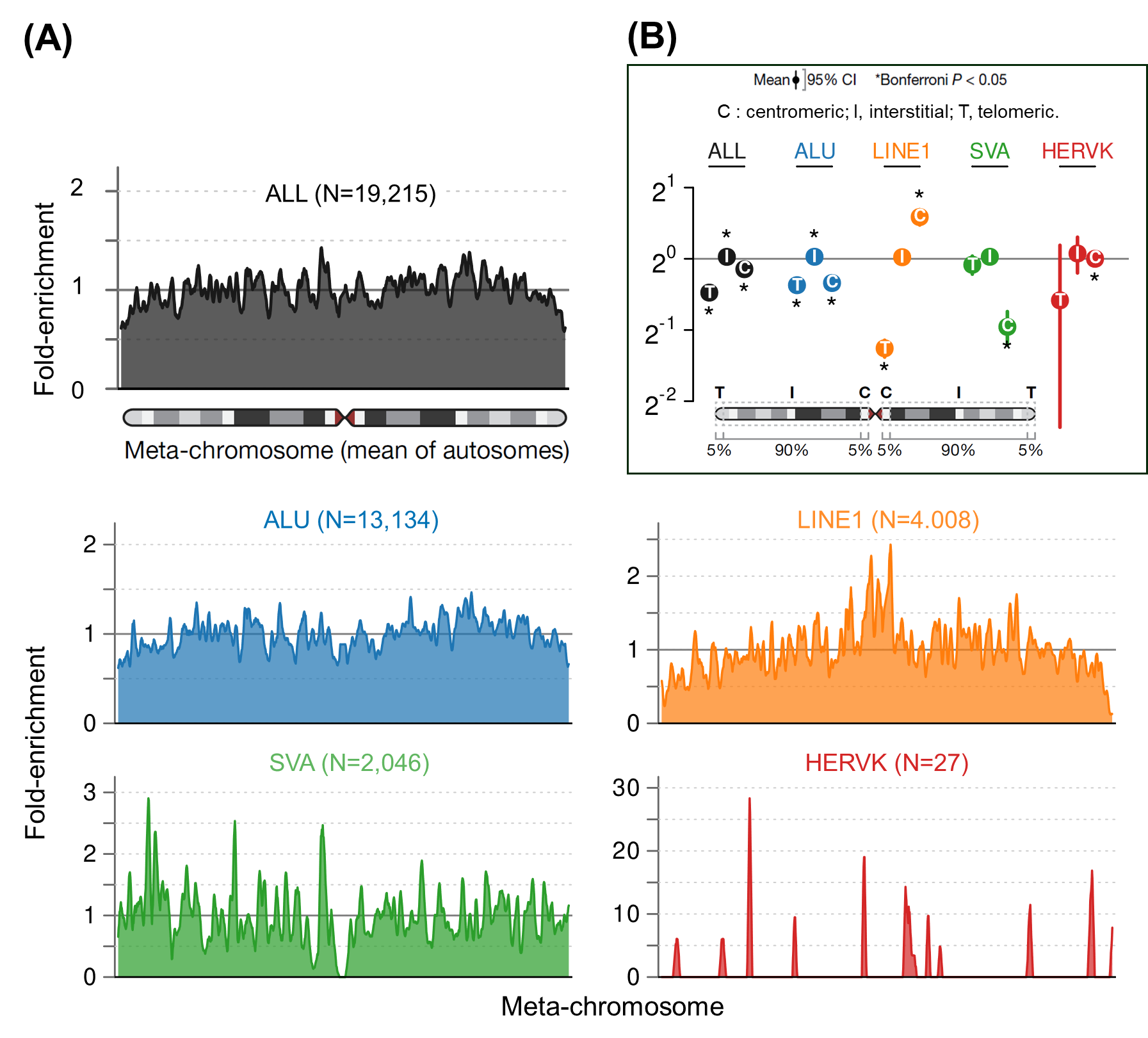


**Suppl Figure 4. Chromosome-level distribution of TE density**. (A) Smoothed enrichment of different types of TEs ascertained in this study. The values were calculated per 100 kb window across the average of all autosomes and normalized by the length of chromosome arms (as ‘meta-chromosome’). (B) Enrichment of MEIs by class and chromosomal context. The dots are the mean values and point ranges represent 95% confidence intervals (CIs). P-values were computed using a two-sided t-test and adjusted using the Bonferroni method. *P ≤ 0.05. C, centromeric; I, interstitial; T, telomeric. The way to compute the chromosomal enrichment and to represent data was from the gnomAD SV paper (Collin et al).**
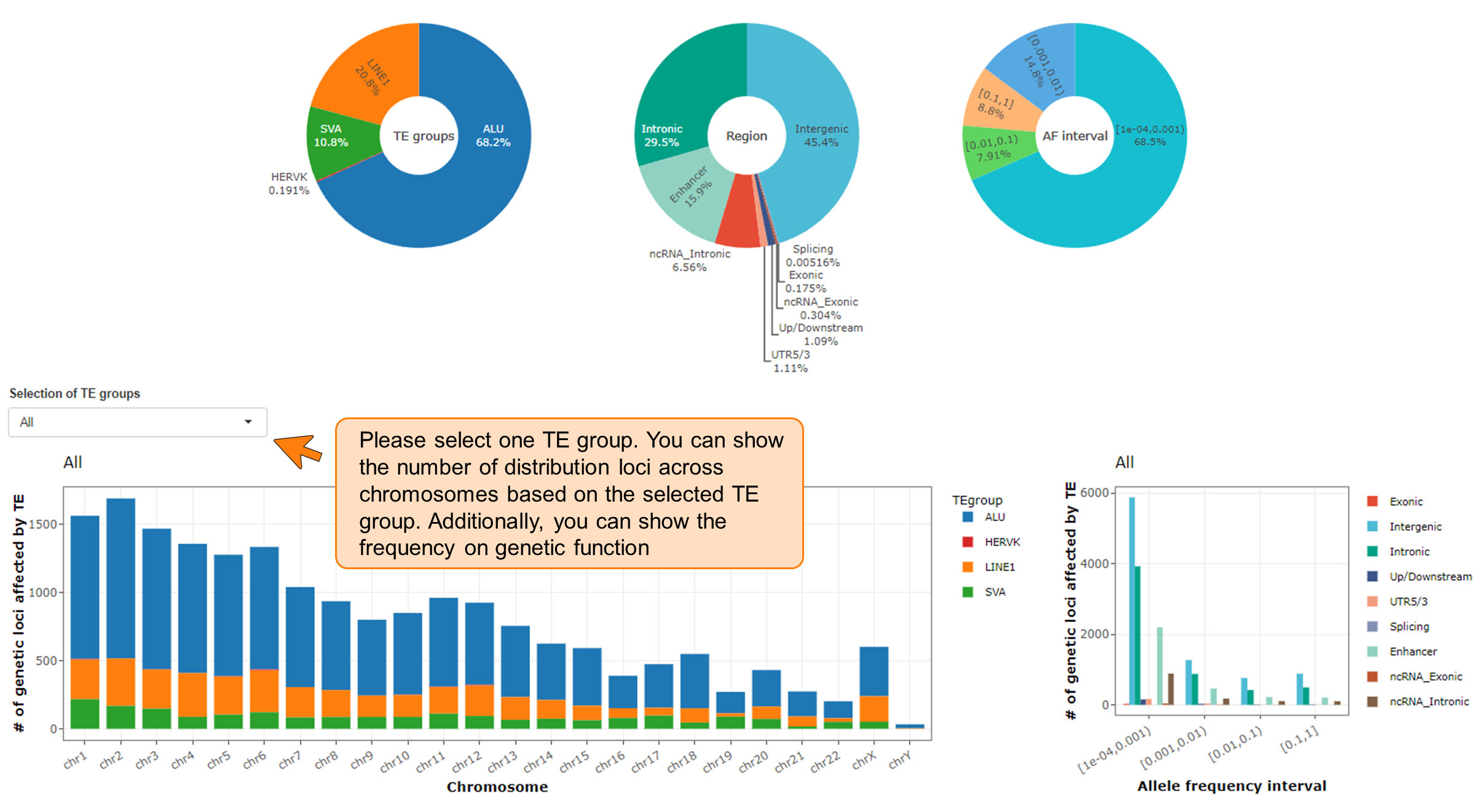
**

**Suppl Figure 5. The distribution of TEs in the ‘Home’**. KTED display the plot of chromosome-wise distribution based on the selected group and the frequency of AF intervals according to the insertion regions within genes.

**
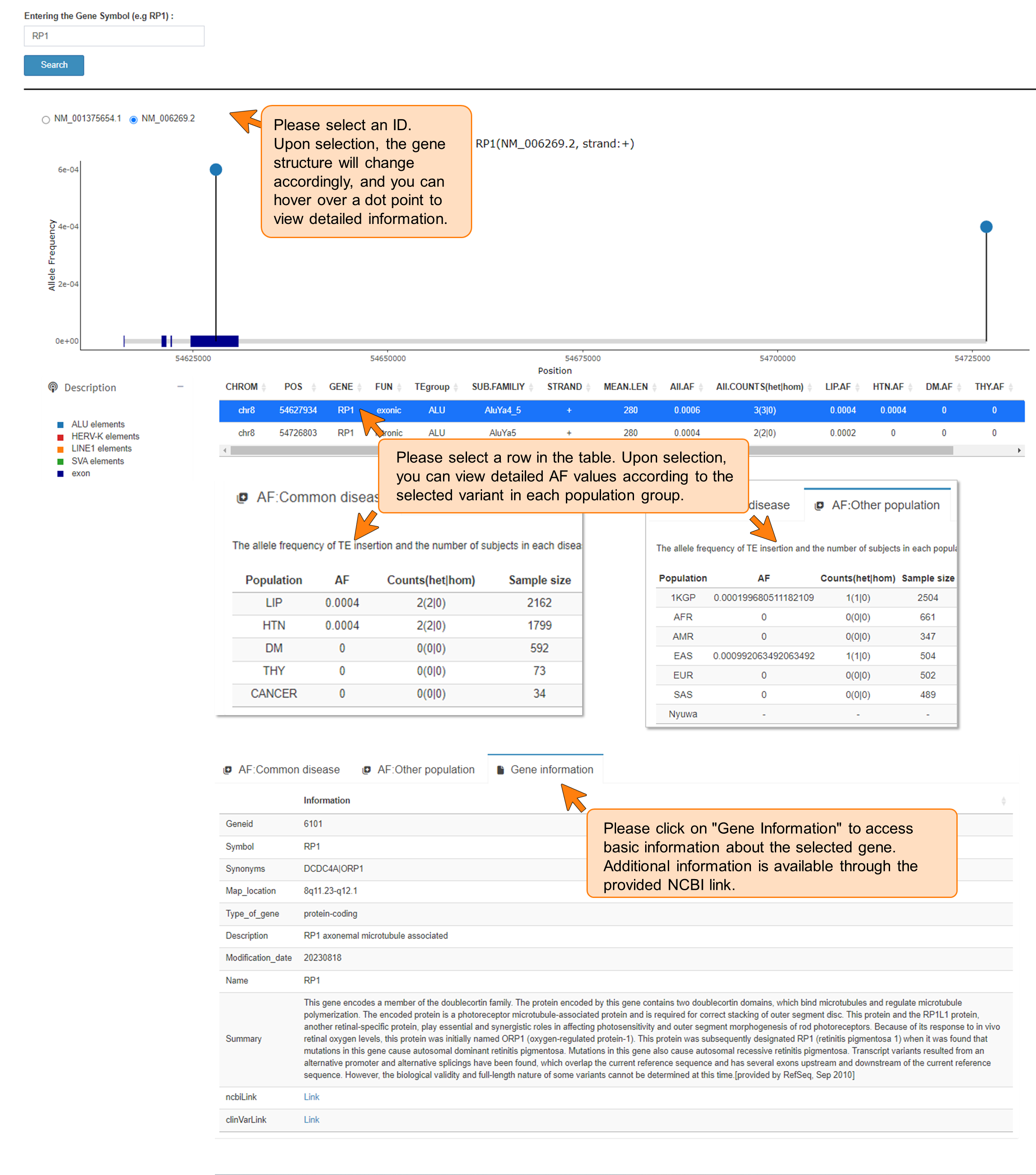
**

**Suppl Figure 6. Example in the ‘Gene search’ menu.**

**
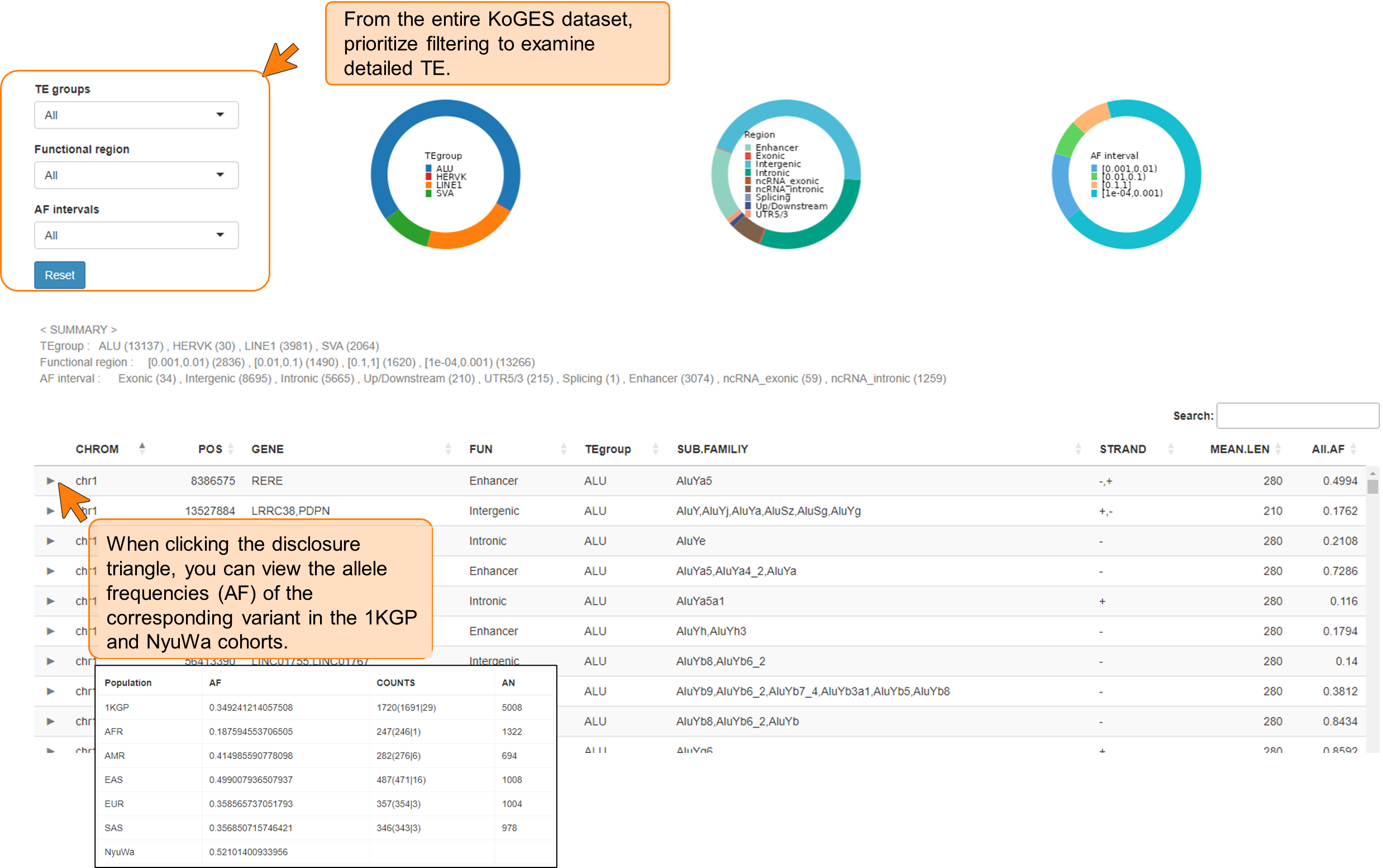
**

**Suppl Figure 7. Example in the ‘Explore’ menu.**
